# Awareness of Vasectomy Among Males​​​​​​: Understanding Knowledge, Attitudes, and Barriers to Adoption in Family Planning

**DOI:** 10.7759/cureus.106716

**Published:** 2026-04-09

**Authors:** Mahendra Gangadharaiah, Srividya Punyala, Punyashree Manchadas

**Affiliations:** 1 Obstetrics and Gynaecology, Adichunchanagiri Institute of Medical Sciences, BG Nagara, IND; 2 Obstetrics and Gynaecology, Sri Chamundeshwari Medical College, Hospital and Research Institute, Ramnagara, IND

**Keywords:** barriers to acceptance, family planning, knowledge and attitudes, male contraception, vasectomy

## Abstract

Introduction

Vasectomy is a safe, simple, and highly effective permanent method of male contraception. Despite its advantages, its utilization remains extremely low in many low- and middle-income countries, including India, where family planning practices remain largely female-centered. Limited awareness, misconceptions regarding sexual performance, and sociocultural barriers contribute to the low acceptance of vasectomy. However, there is limited comprehensive evidence assessing the awareness, knowledge, attitudes, and acceptance (AKAP) framework among men in this setting. The present study aimed to primarily assess the awareness and knowledge of vasectomy, and secondarily to evaluate attitudes, barriers, and factors influencing its acceptance as a method of family planning.

Materials and methods

A hospital-based cross-sectional observational study was conducted in the Department of Obstetrics and Gynecology at Adichunchanagiri Institute of Medical Sciences, B.G Nagara, Karnataka, India, over a period of three months. A total of 500 men aged 18-50 years were included using a convenience sampling technique. Data were collected using a pre-designed, pilot-tested structured questionnaire through face-to-face interviews, covering sociodemographic characteristics, awareness and knowledge regarding vasectomy, attitudes toward the procedure, myths and misconceptions, perceived barriers, and willingness to accept vasectomy. Knowledge and attitude were assessed using composite scoring methods with predefined criteria. Data were analyzed using descriptive statistics, and associations between categorical variables were assessed using the Chi-square test, with statistical significance set at p < 0.05.

Results

Overall awareness of vasectomy was observed in 325 (65%) participants, while adequate knowledge was present in 225 (45%). Only 191 (38%) participants demonstrated a positive attitude toward vasectomy, and willingness to undergo the procedure was reported by 100 (20%) respondents, highlighting a marked decline across the awareness-knowledge-acceptance continuum. Misconceptions were common, with many participants believing that vasectomy causes sexual weakness, impotence, or requires major surgery. Fear of surgery, concerns regarding masculinity, social stigma, and family opposition emerged as major barriers to acceptance. Health workers were identified as the most common source of information among those aware of vasectomy.

Conclusions

Although awareness of vasectomy was moderate, knowledge, positive attitudes, and willingness to accept the procedure were considerably lower, indicating a substantial awareness-acceptance gap. The findings underscore the influence of sociocultural factors and misinformation on male participation in family planning. Addressing misconceptions through targeted, male-focused educational interventions, community-based awareness programs, and structured counseling by healthcare providers may help improve acceptance and promote shared responsibility in family planning.

## Introduction

Vasectomy is a permanent method of male contraception that involves the occlusion of the vas deferens to prevent sperm from entering the ejaculate [1]. It is recognized globally as a safe, simple, minimally invasive, and highly effective procedure, with a failure rate of less than 1% [2]. Importantly, vasectomy does not interfere with sexual performance, hormonal balance, or masculinity, and modern techniques such as no-scalpel vasectomy have further reduced operative time, complications, and the recovery period [3]. Despite these advantages, vasectomy remains one of the least utilized methods of contraception in many low- and middle-income countries [4].

Globally, the uptake of vasectomy varies widely, with higher acceptance observed in certain high-income settings, while participation remains limited in many parts of Asia and Africa [5]. In India, family planning services have historically been female-centered [6]. According to data from the National Family Health Survey (NFHS-5), female sterilization constitutes the predominant method of permanent contraception, whereas vasectomy accounts for less than 1% of total contraceptive use [6,7]. This striking disparity reflects minimal male involvement in reproductive decision-making and highlights the persistent gender imbalance in family planning responsibilities [8].

The low uptake of vasectomy in India, particularly in rural settings, is influenced by multiple factors [9]. Poor awareness, widespread myths regarding physical weakness, fear of reduced sexual performance, misconceptions about impotence, and anxiety related to surgery significantly deter men from considering the procedure [9]. Deep-rooted sociocultural norms often perceive contraception as a woman’s responsibility, further discouraging male participation [10]. Additionally, inadequate counseling, limited promotion of no-scalpel vasectomy, and insufficient engagement of frontline health workers contribute to the persistence of misinformation and low acceptance rates [11].

Understanding the level of awareness, knowledge gaps, prevailing attitudes, and sociocultural barriers among men is essential for designing targeted educational and counseling interventions [12]. Strengthening the role of healthcare providers in disseminating accurate information, along with community-based awareness initiatives involving men, families, and local leaders, may help dispel misconceptions and promote shared responsibility in family planning [8].

However, existing studies have largely evaluated awareness or attitudes in isolation, with limited evidence integrating awareness, knowledge, attitudes, and acceptance using a comprehensive framework, particularly in the Indian context and in hospital-based settings. Addressing this gap is important for developing targeted and context-specific interventions. Therefore, the present study aimed to assess the level of awareness and knowledge regarding vasectomy among men, to evaluate attitudes and willingness to accept vasectomy as a method of family planning, and to identify sociocultural and perceived barriers influencing its uptake.

## Materials and methods

The present study was designed as a hospital-based cross-sectional observational study conducted in the Department of Obstetrics and Gynecology at the Adichunchanagiri Institute of Medical Sciences, B.G. Nagara, Karnataka, India. The study was carried out over a period of three months from December 2025 to February 2026. A hospital-based approach was adopted for feasibility and accessibility to a diverse group of participants, including both patients and attendants from varied sociodemographic backgrounds. Prior approval was obtained from the Institutional Ethics Committee of the institution before the commencement of the study (IEC: AIMS/IEC/277/2025). Confidentiality of participants was maintained throughout, and no personal identifiers were recorded in the data collection forms. Participation was voluntary, and respondents were informed of their right to withdraw from the study at any stage without any consequences.

The sample size for the present cross-sectional study was calculated using the single population proportion formula: 

$n = \frac{Z^2 \times p \times q}{d^2}$

​​​where n is the required sample size, Z is the standard normal deviate at 95% confidence level (CI) (1.96), p is the anticipated prevalence, q = 1 - p, and d is the absolute precision. Based on a previous study by Nair et al. [13], which reported that 70.2% of men were aware of vasectomy, the anticipated prevalence (p) was taken as 0.702, with q = 0.298 and d = 0.05. Substituting these values into the formula yielded a minimum sample size of approximately 322 participants.

A total of 500 males aged 18-50 years were included in the study. Both married and unmarried men attending the hospital, either as patients or accompanying attendants, were approached consecutively during the study period. Participants were recruited using a convenience sampling technique until the required sample size was achieved. While convenience sampling facilitated rapid recruitment, it may limit representativeness and introduce selection bias, which has been considered during the interpretation of the results. Individuals who were willing to participate and provided informed written consent were included. Men with pre-existing medical conditions that contraindicated participation, those unwilling to participate, and men who had already undergone a vasectomy were excluded from the study.

Data were collected using a pre-designed, structured questionnaire administered through face-to-face interviews. The questionnaire used in this study was self-developed by the authors after an extensive review of the relevant literature on vasectomy awareness, knowledge, and attitudes. It was designed to suit the objectives of the study and the local sociocultural context. The questionnaire was pilot-tested on 30 participants to assess clarity, comprehension, and feasibility, and minor modifications were made accordingly before final data collection. However, formal validation and reliability testing (e.g., Cronbach’s alpha) were not performed. As this was a self-structured questionnaire, the complete tool has been included in the Appendix section of the manuscript.

The questionnaire consisted of sections assessing sociodemographic characteristics (age, education, residence, occupation), awareness of vasectomy, detailed knowledge regarding the procedure (permanence, effectiveness, no-scalpel technique, and impact on sexual performance), attitudes toward vasectomy, myths and misconceptions, perceived sociocultural barriers, and willingness to accept the procedure. Knowledge was evaluated using multiple items, and a composite scoring system was applied to categorize participants into good, moderate, and poor knowledge levels based on the proportion of correct responses. Participants with ≥50% correct responses were classified as having “adequate knowledge,” while those with <50% were considered to have “inadequate knowledge.” Attitude toward vasectomy was assessed using structured statements and categorized as positive, neutral, or negative based on response patterns. These categorizations were based on author-defined criteria rather than standardized, validated scales. Face-to-face interviews may have introduced social desirability bias, which was minimized by ensuring confidentiality and a non-judgmental interview approach.

Data were entered into Microsoft Excel and analyzed using appropriate statistical software. Descriptive statistics were expressed as frequencies and percentages. The association between categorical variables such as age, education, attitude, and willingness to accept vasectomy was analyzed using the Chi-square test. A p-value of less than 0.05 was considered statistically significant. Multivariate analysis (e.g., logistic regression) was not performed due to the descriptive scope of the study; however, future studies are recommended to explore adjusted associations and control for potential confounders.

## Results

The study included 500 participants with varied sociodemographic characteristics (Table 1).

**Table 1:** Sociodemographic characteristics of the participants (N = 500)

Variable	Category	N (%)
Age group, years	20–29	200 (40%)
	30–39	150 (30%)
	40–49	150 (30%)
Educational status	Primary	110 (22%)
	Secondary	210 (42%)
	Graduate and above	180 (36%)
Residence	Urban	290 (58%)
	Rural	210 (42%)
Occupation	Skilled	210 (42%)
	Unskilled	170 (34%)
	Professional	120 (24%)

The awareness of vasectomy in the cohort was moderate, but accurate knowledge was lower, indicating a knowledge gap (Table 2).

**Table 2:** Awareness and knowledge regarding vasectomy (N = 500)

Variable	N (%)
Heard about vasectomy	325 (65%)
Correct overall knowledge	225 (45%)
Aware that vasectomy does not affect sexual performance	210 (42%)
Aware of no-scalpel vasectomy	180 (36%)
Aware that vasectomy is a permanent method	240 (48%)
Aware that vasectomy is highly effective (>99%)	195 (39%)

Attitudes towards differed significantly across age groups, with younger men showing relatively more positive attitudes (Table 3).

**Table 3:** Attitude towards vasectomy by age group (N = 500) Statistical analysis was performed using the Chi-square test to determine the association between age group and attitude toward vasectomy. A p-value < 0.05 was considered statistically significant

Age group, years	Positive	Neutral	Negative	Total	Chi-square value	P-value
20–29 (n = 200)	110 (55%)	70 (35%)	20 (10%)	200	69.8021	< 0.0001
30–39 (n = 150)	51 (34%)	68 (45%)	31 (21%)	150		
40–49 (n = 150)	30 (20%)	57 (38%)	63 (42%)	150		
Total (N = 500)	191 (38%)	195 (39%)	114 (23%)	500		

Health workers were the main source of information among those aware of vasectomy (Table 4).

**Table 4:** Source of information about vasectomy among those who are aware (n = 325)

Source of information	N (%)
Health workers	221 (68%)
Mass media	72 (22%)
Peers/friends	26 (8%)
Other sources	6 (2%)

Misconceptions regarding vasectomy were highly prevalent, particularly related to sexual performance, physical strength, and the nature of the procedure (Table 5).

**Table 5:** Myths and misconceptions regarding vasectomy (N = 500)

Misconception	N (%)
Causes sexual weakness	240 (48%)
Leads to impotence	210 (42%)
Reduces physical strength/work capacity	175 (35%)
Requires major surgery and long hospitalization	260 (52%)
Causes permanent disability	150 (30%)

Major barriers to acceptance included fear of surgery, sociocultural factors such as concerns about masculinity and stigma, and opposition from family members (Table 6).

**Table 6:** Perceived barriers to acceptance of vasectomy (N = 500)

Barrier	N (%)
Fear of surgery	290 (58%)
Fear of complications	210 (42%)
Misconceptions about masculinity	240 (48%)
Social stigma	240 (48%)
Family opposition	185 (37%)

Willingness to accept vasectomy was low and showed a significant association with attitude, with acceptance being higher among those with positive attitudes (p < 0.0001) (Table 7).

**Table 7:** Attitude in relation to willingness to accept vasectomy (N = 500) The association between attitude and willingness was assessed using the Chi-square test. A p-value < 0.05 was considered statistically significant

Attitude	Willing, n (%)	Not willing, n (%)	Total	Chi-square (χ²)	P-value
Positive	75 (39%)	116 (61%)	191	73.2552	< 0.0001
Neutral	20 (10%)	175 (90%)	195		
Negative	5 (4%)	109 (96%)	114		
Total	100 (20%)	400 (80%)	500		

A progressive decline was observed across the awareness-knowledge-acceptance continuum, highlighting a clear awareness-acceptance gap (Table 8).

**Table 8:** Awareness-knowledge-acceptance gap (AKAP model) (N = 500) AKAP: awareness-knowledge-attitude-practice/acceptance model

Component	N (%)
Awareness	325 (65%)
Adequate knowledge	225 (45%)
Positive attitude	191 (38%)
Willingness to accept	100 (20%)

## Discussion

The present study assessed awareness, knowledge, attitudes, and barriers related to vasectomy among men and revealed a substantial gap between awareness and actual acceptance of the procedure. In this study, 325 (65%) participants had heard about vasectomy; however, only 225 (45%) demonstrated adequate knowledge, and merely 100 (20%) were willing to undergo the procedure. This decline across the awareness-knowledge-acceptance continuum suggests that awareness alone is insufficient to translate into acceptance of male sterilization. This observed gap can be explained by the persistence of misconceptions, sociocultural norms, and inadequate risk perception despite baseline awareness. Ayele et al. conducted a community-based study among married men in Ethiopia and found that only 223 (38.5%) participants had good knowledge regarding vasectomy, indicating that inadequate knowledge remains a major factor limiting the utilization of this contraceptive method [14].

In the present study, knowledge regarding specific aspects of vasectomy was limited. Only 180 (36%) participants were aware of the no-scalpel vasectomy technique, and 195 (39%) knew that vasectomy is highly effective in preventing pregnancy. These findings are comparable to those reported in other studies conducted in developing countries. Wolde et al. reported that although approximately half of the participants had heard about vasectomy, only a smaller proportion demonstrated adequate knowledge about its safety, effectiveness, and procedural details [15]. Similar findings have been reported in India by Ganesh R. Nair et al., who observed that although 70.2% of men were aware of vasectomy, accurate knowledge and favorable attitudes were considerably lower, highlighting persistent knowledge gaps and gender bias in contraceptive practices [13].

Attitudes toward vasectomy in the present study varied significantly across age groups, with younger men demonstrating comparatively more positive attitudes. Overall, 191 (38%) participants showed a positive attitude toward vasectomy, while a considerable proportion remained neutral or negative. Hurisa et al. reported that although more than half of the respondents had favorable attitudes toward vasectomy, the actual intention to adopt the method remained relatively low, highlighting the persistent discrepancy between positive perceptions and practical acceptance [16]. The relatively more favorable attitude among younger men in the present study may be attributed to better exposure to education, media, and evolving gender norms, whereas older men may be more influenced by traditional beliefs, cultural expectations, and resistance to change.

Misconceptions regarding vasectomy were highly prevalent in the present study and constituted a major barrier to its acceptance. Nearly half of the participants believed that vasectomy causes sexual weakness (240, 48%) or impotence (210, 42%), and more than half perceived that it requires major surgery and prolonged hospitalization (260, 52%). Similar misconceptions have been documented in other studies. Shrivastava et al., in a community-based study conducted in South India, also reported that myths related to sexual dysfunction, loss of masculinity, and reduced physical strength significantly influenced men’s reluctance to adopt vasectomy [17]. These findings are strongly supported by the systematic review by Koushik Biswas et al., which identified key barriers in the Indian context, including fear of surgery, concerns about loss of libido, perceived physical weakness, social stigma, and cultural beliefs linking masculinity with fertility [18]. This highlights the deep-rooted sociocultural constructs of masculinity and societal expectations that significantly hinder male participation in family planning.

Another important observation in the present study was that health workers were the main source of information for 221 (68%) participants who had heard about vasectomy, followed by mass media and peers. This finding underscores the crucial role of healthcare providers in disseminating accurate information regarding family planning methods. Similar findings have been reported by White et al., who highlighted that healthcare professionals play a key role in improving men’s knowledge and attitudes toward vasectomy through counseling and educational interventions [19]. In the Indian context as well, strengthening behavior change communication and counseling strategies has been emphasized as essential to improve male participation in family planning, as highlighted by Nair et al. [13].

Strengths and limitations

The present study has several strengths. It included a relatively large sample size of 500 participants, which enhances the reliability and generalizability of the findings within the study population. The study comprehensively evaluated multiple dimensions of vasectomy awareness, including knowledge, attitudes, misconceptions, sources of information, and barriers to acceptance, thereby providing a holistic understanding of male perspectives toward vasectomy. Additionally, the use of the awareness, knowledge, attitudes, and acceptance (AKAP) framework allowed for a systematic assessment of the gap between awareness and acceptance of vasectomy.

However, certain limitations should also be considered while interpreting the findings. As a cross-sectional study, causal relationships between awareness, attitudes, and willingness to adopt vasectomy cannot be established. The study relied on self-reported responses, which may be subject to recall bias and social desirability bias. Furthermore, the study population was limited to a specific geographic area, which may restrict the generalizability of the results to other regions with different sociocultural contexts. Additionally, the use of convenience sampling may have introduced selection bias, and the hospital-based design may limit the representativeness of the general population. The questionnaire used was self-structured and not formally validated, which may affect measurement reliability. Moreover, the absence of multivariate analysis limits the ability to control for potential confounding variables such as education, residence, and occupation.

## Conclusions

The present study demonstrates that although awareness of vasectomy among men was moderate, accurate knowledge, positive attitudes, and willingness to adopt the procedure were considerably lower, indicating a substantial awareness-acceptance gap. Misconceptions regarding sexual weakness, impotence, and the belief that vasectomy requires major surgery were common and emerged as major barriers to acceptance. Fear of surgery, social stigma, and concerns related to masculinity further limited men’s willingness to consider vasectomy as a contraceptive option. Health workers were identified as the primary source of information, highlighting their crucial role in disseminating accurate knowledge and promoting male participation in family planning. The findings underscore the need for targeted, male-focused interventions to bridge the gap between awareness and acceptance. Therefore, strengthening educational initiatives, including structured behavior change communication (BCC) strategies, community awareness programs, and culturally sensitive counseling strategies, is essential to dispel myths, improve knowledge, and enhance acceptance of vasectomy as a safe, effective, and reliable method of contraception. In addition, integrating vasectomy counseling into routine primary healthcare services, actively involving male partners in family planning discussions, and training frontline health workers to address misconceptions can significantly improve uptake. Future multicenter and community-based studies using validated tools and advanced analytical methods are recommended to further explore determinants of vasectomy acceptance and guide evidence-based policy interventions.

## Appendices

Case Record Form (CRF)

Title of the study: Awareness of Vasectomy Among Males​​​: Understanding Knowledge, Attitudes, and Barriers to Adoption in Family Planning

Participant ID: __________________

Date of Interview: __________________

Place of Interview: __________________

Interviewer Name: __________________

Section A: sociodemographic details

Age (years): __________________

Age Group (20-29 / 30-39 / 40-49): __________________

Educational Status (Primary / Secondary / Graduate and above): __________________

Residence (Urban / Rural): __________________

Occupation (Skilled / Unskilled / Professional): __________________

Section B: awareness and knowledge regarding vasectomy

Have you ever heard about vasectomy? Response: __________________

Do you know that vasectomy does not affect sexual performance? Response: __________________

Are you aware of the no-scalpel vasectomy technique? Response: __________________

Do you know that vasectomy is a permanent method of contraception? Response: __________________

Are you aware that vasectomy is highly effective (more than 99%) in preventing pregnancy? Response: __________________

Overall knowledge regarding vasectomy (adequate/inadequate): __________________

Section C: attitude towards vasectomy

What is your overall attitude toward vasectomy as a family planning method? Response: __________________ (Positive / Neutral / Negative)

Section D: source of information about vasectomy

(To be filled only if the participant has heard about vasectomy)

What was your main source of information about vasectomy? Response: __________________ (Health worker / Mass media / Peers or friends / Other)

Section E: myths and misconceptions regarding vasectomy

Do you believe vasectomy causes sexual weakness? Response: __________________

Do you believe vasectomy leads to impotence? Response: __________________

Do you believe vasectomy reduces physical strength or work capacity? Response: __________________

Do you believe vasectomy requires major surgery and prolonged hospitalization? Response: __________________

Do you believe vasectomy can cause permanent disability? Response: __________________

Section F: perceived barriers to acceptance of vasectomy

Do you have a fear of surgery regarding vasectomy? Response: __________________

Do you fear complications after vasectomy? Response: __________________

Do you think vasectomy affects masculinity? Response: __________________

Do you think there is a social stigma associated with vasectomy? Response: __________________

Do you think family members would oppose vasectomy? Response: __________________

Section G: willingness to accept vasectomy

Are you willing to undergo vasectomy as a family planning method? Response: __________________
